# Safety and efficacy of the combination simeprevir-sofosbuvir in HCV genotype 1- and 4-mono-infected patients from the French ANRS CO22 hepather cohort

**DOI:** 10.1186/s12879-019-3923-5

**Published:** 2019-04-02

**Authors:** Anne Laurain, Sophie Metivier, Georges Haour, Dominique Larrey, Céline Dorival, Christophe Hezode, Fabien Zoulim, Patrick Marcellin, Marc Bourliere, Jean-Pierre Zarski, Dominique Thabut, Laurent Alric, Nathalie Ganne-Carrie, Paul Cales, Jean-Pierre Bronowicki, Ghassan Riachi, Claire Geist, Xavier Causse, Armand Abergel, Olivier Chazouilleres, Philippe Mathurin, Dominique Guyader, Didier Samuel, Albert Tran, Véronique Loustaud-Ratti, Ventzislava Petrov-Sanchez, Alpha Diallo, Clovis Luzivika-Nzinga, Hélène Fontaine, Fabrice Carrat, Stanislas Pol, on behalf of the ANRS/AFEF HEPATHER study group, Delphine Bonnet, Virginie Sicart, François Bailly, Marjolaine Beaudoin, Dominique Giboz, Kerstin Hartig-Lavie, Marianne Maynard, Morane Cavellec, Marjorie CheraudCarpentier, François Raffi, Florian Vivrel, Eric Billaud, Jaouad Benhida, Jérôme Boursier, Paul Calès, Françoise Lunel, Frédéric Oberti, Isabelle Hubert-Fouchard, Nathalie Boyer, Audrey Gilibert, Nathalie Giuily, Giovanna Scoazec, Sandrine Fernandes, Sylvie Keser, Philippe Sultanik, Anaïs Vallet-Pichard, Juliette Foucher, Jean-Baptiste Hiriart, Aurore Mathias, Julien Vergniol, Chrystelle Ansaldi, Laëtitia Chouquet, Emilie De Luca, Valérie Oules, Rodolphe Anty, Eve Gelsi, Régine Truchi, Elena Luckina, Nadia Messaoudi, Joseph Moussali, Barbara De Dieuleveult, Damien Labarriere, Pascal Potier, Si Nafa Si Ahmed, Xavier Causse, Nathalie Ganne-Carrié, Véronique Grando-Lemaire, Pierre Nahon, Alan Peltier, Judith Ung, Valérie Bourcier, Mariette Gougeon, Anne Guillygomarch, Caroline Jezequel, Romain Moirand, Thomas F. Baumert, Michel Dofföel, Catherine Mutter, Pauline Simo-Noumbissie, François Habersetzer, Hélène Barraud, Mouni Bensenane, Abdelbasset Nani, Sarah Hassani-Nani, Marie-Albertine Bernard, Michael Bismuth, Ludovic Caillo, Stéphanie Faure, Georges-Philippe Pageaux, Marie Pierre Ripault, Karl Barange, Christophe Bureau, Jean Marie Peron, Marie Angèle Robic, Léa Tarallo, Marine Faure, Bruno Froissart, Marie-Noelle Hilleret, Vincent Leroy, Odile Goria, Victorien Grard, Hélène Montialoux, Muriel François, Christian Ouedraogo, Christelle Pauleau, Anne Varault, Tony Andreani, Bénédicte Angoulevant, Azeline Chevance, Lawrence Serfaty, Teresa Antonini, Audrey Coilly, Jean-Charles Duclos Vallée, Mariagrazia Tateo, Armand Abergel, Corinne Bonny, Chanteranne Brigitte, Géraldine Lamblin, Léon Muti, Abdenour Babouri, Virginie Filipe, Camille Barrault, Laurent Costes, Hervé Hagège, Soraya Merbah, Paul Carrier, Maryline Debette-Gratien, Jérémie Jacques, Florent Artu, Valérie Canva, Sébastien Dharancy, Alexandre Louvet, Marc Bardou, Donya Da Costa Souhiel, Patrick Hillon, Marianne Latournerie, Yannick Bacq, Didier Barbereau, Charlotte Nicolas, Nisserine Ben Amara, Danièle Botta-Fridlund, Isabelle Portal, Moana Gelu-Simeon, Marie-Josée Lafrance

**Affiliations:** 1 Université Paris Descartes ; APHP, Unité d’Hépatologie, Hôpital Cochin ; INSERM U-818 et USM20, Institut Pasteur, Paris, France; 2 Department of Hepatology and Gastroenterology, CHU Purpan, Toulouse, France; 3 Sorbonne Université, INSERM, Institut Pierre Louis d’épidémiologie et de Santé Publique, F75012 Paris, France; 4 Liver unit-IRB-INSERM1040, Hôpital Saint Eloi, Montpellier, France; 5 Department of Hepatology and Gastroenterology, Hôpital Henri Mondor, AP-HP, Université Paris-Est, INSERM U955, Créteil, France; 6 Department of Hepatology, Hospices Civils de Lyon, INSERM U1052, Université de Lyon, Lyon, France; 7 Department of Hepatology, Hôpital Beaujon, AP-HP, Université Paris-Diderot, INSERM CRB3, Clichy, France; 8 Department of Hepatology and Gastroenterology, Hôpital Saint Joseph, Marseille, France; 9 Department of Hepatology and Gastroenterology, Centre Hospitalo-Universitaire, INSERM U823, Grenoble, France; 10 Department of Hepatology and Gastroenterology, Groupe Hospitalier Pitié-Salpétrière, AP-HP, Université Pierre et Marie Curie Paris 6, INSERM UMR-S938, Paris, France; 11 Internal Medicine-Digestive Department CHU Purpan, UMR152, IRD, Toulouse 3 University, Toulouse, France; 12 Functional Genomics of Solid Tumors, Hepatology Unit, Hôpital Jean Verdier, Bondy, AP-HP, University Paris 13, Sorbonne Paris Cité, Bobigny; Inserm UMR-1162, F-93000 Paris, France; 13 Liver-Gastroenterology Department, CHU Angers, Angers, France; 14 Department of Hepatology and Gastroenterology, Centre Hospitalier Universitaire de Nancy, Université de Lorraine, INSERM U954, Vandoeuvre-les-Nancy, France; 15 Department of Hepatology and Gastroenterology, CHU Charles Nicolle, Rouen, France; 16 Department of Hepatology and Gastroenterology, Centre Hospitalier Régional, Metz, France; 17 Department of Hepatology and Gastroenterology, CHR d’Orléans, Orléans, France; 18 Department of Digestive and Hepatobiliary Diseases, Estaing University Hospital, Clermont-Ferrand, France; 19 UMR Auvergne University/CNRS 6284 ISIT (Image Sciences for Innovations Techniques), Clermont-Ferrand, France; 20 Department of Hepatology, Hôpital Saint-Antoine, AP-HP, Université Pierre et Marie Curie Paris 6, Paris, France; 21 Department of Hepatology and Gastroenterology, Centre Hospitalier Régional et Universitaire Claude Huriez, Lille, France; 22 Liver disease unit, CHU Rennes, Université de Rennes 1, INSERM U991, Rennes, France; 23 Centre Hépato-Biliaire, Hôpital Paul Brousse, AP-HP, UMR-S785, Université Paris-Sud, INSERM U785, Villejuif, France; 24 Digestive Center, Centre Hospitalier Universitaire de Nice, INSERM U1065-8, Nice, France; 25 Department of Hepatology and Gastroenterology, CHU Limoges, U850 INSERM, Univ. Limoges, F-87000 Limoges, France; 26 ANRS (France Recherche Nord&sud Sida-hiv Hépatites), Unit for Basic and Clinical Research on Viral Hepatitis, Paris, France; 27 ANRS (France Recherche Nord&sud Sida-hiv Hépatites), Clinical Trial Safety and Public Health, Paris, France; 28 Assistance Publique-Hôpitaux de Paris, Hôpital Saint Antoine, Unité de Santé Publique, F-75012 Paris, France

**Keywords:** Direct acting antivirals, Hepatitis C virus, Real life cohort, Simeprevir, Sofosbuvir

## Abstract

**Background:**

Although real-life results of sofosbuvir/simeprevir have been extensively reported from the United States, data from other geographical areas are limited. In the French observational cohort, ANRS CO22 HEPATHER, 9432 patients were given the new oral antivirals from December 2013 to June 30, 2018. We report the results of sofosbuvir/simeprevir in genotypes 1- and 4-infected patients.

**Methods:**

Demographics and history of liver disease were collected at entry in the cohort. Clinical, adverse events, and virological data were collected throughout treatment and post-treatment follow-up. The choice of treatment duration or addition of ribavirin was left up to the physician.

**Results:**

Five hundred ninety-nine HCV (467 genotype 1 and 132 genotype 4) mono-infected, naïve for all oral-DAAs regimen patients were given sofosbuvir/simeprevir with (*n* = 63) or without ribavirin (*n* = 536) for 12 or 24 weeks; 56% had cirrhosis (4% decompensated) and 71% had prior treatment failure to interferon-based regimen. 7 patients (1.16%) were lost to follow-up. The overall SVR12 rate was 92.6%. The SVR12 was 90% in GT1a, 94.2% in GT1b and 91.6% in GT4 with no significant difference for genotype, treatment duration or ribavirin addition. Severity of liver disease was not associated with a lower SVR12 rate on multivariate analysis but was associated with a higher rate of severe side effects. Early treatment discontinuations were rare; no new safety signals were reported.

**Conclusion:**

In this real life, observational, prospective cohort study, the 12-week sofosbuvir/simeprevir+/−ribavirin combination appears to be efficient and safe.

**Trial registration:**

Trial registration with ClinicalTrials.gov NCT01953458.

## Key points

- The combination of sofosbuvir and simeprevir results in an overall SVR12 of 92.6, 90% in patients with genotype 1a infection, 94.2% with genotype 1b and 91.6% with genotype 4.
- The safety of the sofosbuvir and simeprevir combination was acceptable with only 3% of early discontinuations and with no new safety warnings.
- The combination with sofosbuvir and simeprevir is no longer recommended but remains a potential therapeutic option in resource-limited settings or in countries where simeprevir is still available.

## Background

Chronic hepatitis C virus (HCV) infection is a worldwide disease that is responsible for hepatic and extrahepatic morbidity and mortality [1, 2]. A sustained virological response (SVR) corresponds to a complete cure of infection. A SVR is also associated with a reduction in HCV-related complications such as cirrhosis, hepatocellular carcinoma, the need for transplantation and death [3–6]. A better understanding of the viral cycle and characterization of the non-structural proteins of the virus led to development of direct acting antivirals (DAAs) against HCV [7–9]. Approved in the Spring of 2011, second generation DAAs replaced first generation protease inhibitors, which were then removed from the market in 2014. NS5B polymerase inhibitors (sofosbuvir, dasabuvir), protease inhibitors (simeprevir, paritaprevir, grazoprevir, glecaprevir, voxilaprevir) and NS5A replication complex inhibitors (daclatasvir, ledipasvir, ombitasvir, elbasvir, pibrentasvir and velpatasvir) have also been approved and evaluated [10–21]. A combination of pangenotypic drugs is now recommended to treat chronic HCV infection, while EASL guidelines no longer recommend the combination of sofosbuvir and simeprevir [22–24]. For the European Association for the Study of the Liver (EASL), antiviral therapy should be considered in all patients with chronic HCV infection because of the efficacy and safety profile of DAAs. Because of the long timelines for approval and in addition to clinical trials, preliminary real-life results of the combination of sofosbuvir/simeprevir have been extensively reported [25–30] from the United States (US) in patients with genotype 1 infection. However, real-life data from Europe or outside the US, and for genotype 4 are limited [31–33].

We report the real-life results of the French ANRS CO22 Hepather cohort for the sofosbuvir+simeprevir +/− ribavirin combination in patients with HCV genotypes 1 or 4 mono-infection.

## Methods

### Study design and participants

The ANRS CO22 HEPATHER cohort « Therapeutic option for hepatitis B and C: a French cohort » is a multicenter, national, prospective, observational cohort study of patients infected with hepatitis B or C virus (ClinicalTrials.gov registry number: NCT01953458). The cohort has been extensively described elsewhere [34].

In summary, by December 31, 2015, 20,798 patients had been included in the cohort, including 14,195 HCV-positive patients. A total of 9432 patients were given treatment including at least one direct acting antiviral from December 2013 to June 30, 2018. We selected all patients with HCV genotype 1 or genotype 4 infection who initiated a combination of sofosbuvir (400 mg/d) and simeprevir (150 mg/d) with or without ribavirin (1–1.2 g/d) before October 31, 2014 (*n* = 599). Patients who were liver transplant recipients, previously treated with other DAAs (except first generation protease inhibitors) or involved in clinical trials, were excluded. Patients were divided into four groups according to the scheduled duration of treatment and whether the regimen included ribavirin. This was an observational and not a randomized controlled study and the choice of treatment combination, duration and addition of ribavirin was left up to the physician. The diagnosis of cirrhosis was based either on the results of liver biopsy, a fibrotest result > 0.7 or fibroscan greater than 14.5 kPa. The duration of chronic hepatitis was estimated by the date of contamination, if available.

### Outcomes

The main endpoint criterion was SVR at 12 weeks (SVR12) defined as undetectable HCV RNA 12 weeks after the last treatment. Secondary endpoints were undetectable HCV RNA 4 weeks after the last treatment (SVR4), early treatment discontinuation and adverse events.

### Statistical analyses

A post-hoc calculation showed that the present study achieved a reliability of 2.4% for an anticipated 90% SVR12 and had a power > 80% for detecting Odds-Ratio (OR) < 0.3 for factors associated with SVR12, assuming the exposure to these factors ranged between 20 to 75%. Missing SVR12 measurements were determined using SVR24 measurements, if available (*n* = 52), otherwise using the SVR4 measurement (*n* = 6). Patients who died before SVR12 and therefore could not be evaluated (*n* = 4) and patients who were lost of follow-up (*n* = 7) and had no measurement of the virological response after the end of therapy, were considered to be virological failures.

Proportions were compared using the Fisher exact test and/or continuous outcomes were compared using the Kruskal-Wallis test. Comparisons with stratification for treatment duration or ribavirin use were tested using the Cochran-Mantel Haenszel (CMH) Chi-Square. Exact logistic regression models were used to assess independent baseline variables associated with SVR12 or serious side effects (including death). Predefined cut-offs were used to categorize all continuous factors. A univariate exact logistic model was estimated for each factor. Primary multivariate analysis included ribavirin (No vs Yes), treatment duration (12 vs. 24 weeks) and any factors with a *P*-value < 0∙10 on univariate analysis. A backward selection was applied retaining variables with a P-value < 0∙05. Statistical analyses were performed with SAS 9.4 software (SAS Institute Inc., Cary, North Carolina, USA).

### Role of the funding source

The ANRS CO22 HEPATHER cohort is sponsored by Inserm-ANRS (French National Institute for Health and Medical Research – ANRS/France REcherche Nord&Sud Sida-hiv Hépatites). The sponsor contributed to the study design and writing of this report. The sponsor had no role in data collection, data analysis or data interpretation. The other sponsors of the study had no role in study design, data collection, data analysis, data interpretation, or writing of the report. FC had full access to all the data in the study and SP and FC had the final responsibility for the decision to submit for publication.

## Results

### Patient population

**Fig. 1:**
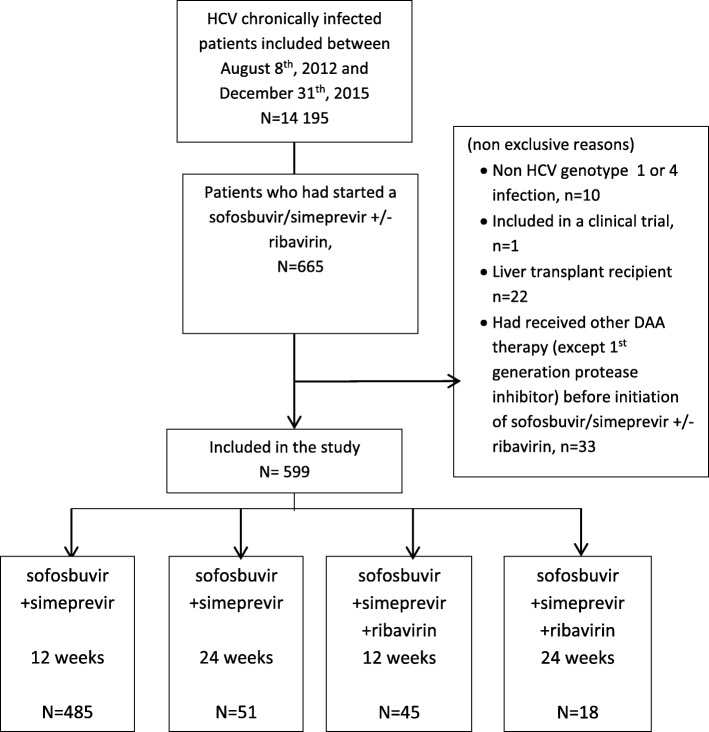
Flow diagram

By October 31, 2014, 599 cohort participants with HCV genotypes 1 (*N* = 467) or genotype 4 (*N* = 132) infection had started treatment with the sofosbuvir/simeprevir combination including 536 (89%) who did not receive and 63 (11%) who did receive ribavirin based on the physician’s decision (Fig. 1). The duration of treatment was 12 weeks in 530 (530/599 = 88.4%) patients and 24 weeks in 69 (69/599 = 11.5%).

Patient characteristics are described in Table 1. Fifty-four percent were men, mean age 61 years old, 56% with cirrhosis, 4% with decompensated cirrhosis and 71% with interferon-based treatment failure (all were naïve for all oral DAAs regimens). Patients were infected with genotype 1a (23.5%), 1b (49.5%), 1 but unclassified (5%) or 4 (22%). The mean MELD score in the 312 with available data out of 335 patients with cirrhosis was 9.5 +/− 4.3 and 91.6% were Child-Pugh score A. Patients who received the 12-week combination of sofosbuvir/simeprevir differed from those who received the 24-week or ribavirin-containing regimens, with lower rates of cirrhosis (54% (259/478) vs 67% (76/114), *p* = 0.0158), decompensated cirrhosis (3% (16/485) vs 7% (8/114), *p* = 0.1051) and biochemical markers of liver severity: albumin< 30 g/L in 3% (12/437) vs 7% (7/101), *p* = 0.0650, prothrombin time ≤ 70% in 10% (45/449) vs 21% (23/110), *p* = 0.0031, thrombocyte count < 100,000/mm3 in 19% (87/468) vs 31% (34/111), *p* = 0.064 and conjugated bilirubinemia ≥5 μmol/L in 52% (133/257) vs 67% (49/73), *p* = 0.0233.

**Table 1:** Baseline characteristics of patients

	SOF SMV 12 weeks *n* = 485	SOF SMV 24 weeks *n* = 51	SOF SMV RBV 12 weeks *n* = 45	SOF SMV RBV 24 weeks *n* = 18	*p* value
Age (years), mean ± SD	61 ± 11	60 ± 10	59 ± 11	58 ± 13	0.2908
Gender Male n (%)	256 (53)	24 (47)	28 (62)	14 (78)	0.0866
BMI (kg/m2) n (%)					
≥ 30	74 (15)	10 (20)	7 (16)	2 (11)	0.9531
< 18.5	12 (2)	2 (4)	0 (0)	0 (0)	
[25–30[	163 (34)	14 (27)	17 (38)	7 (39)	
[18.5–25[	233 (48)	25 (49)	21 (47)	9 (50)	
Chronic hepatitis duration (years), mean ± SD	16 ± 8	15 ± 7	16 ± 9	11 ± 8	0.0814
HCV genotype n (%)					
1a	110 (23)	11 (22)	14 (31)	6 (33)	0.2543
4	99 (20)	17 (33)	10 (22)	6 (33)	
1 not subtyped	25 (5)	1 (2)	3 (7)	0 (0)	
1b	251 (52)	22 (43)	18 (40)	6 (33)	
Diabetes n (%)	104 (21)	7 (14)	9 (20)	1 (6)	0.2710
Hypertension n (%)	194 (40)	25 (49)	18 (40)	4 (22)	0.2571
Cirrhosis n (%)	259 (54)	33 (65)	27 (60)	16 (89)	**0.0117**
•Child-Pugh score B or C	17 (7)	8 (24)	1 (4)	2 (13)	**0.0083**
•MELD ≥15	19 (8)	4 (13)	1 (4)	3 (19)	0.2444
•Elastography ≥14.5 kPa	131 (51)	15 (45)	14 (52)	10 (63)	0.7540
•Fibrotest ≥0.73	70 (27)	10 (30)	11 (41)	10 (63)	**0.0175**
•Liver biopsy > 2 years	148 (57)	20 (61)	14 (52)	7 (44)	0.6618
•Liver biopsy < 2 years	18 (7)	2 (6)	1 (4)	0 (0)	0.9306
Decompensated cirrhosis n (%)	16 (3)	6 (12)	2 (4)	0 (0)	**0.0483**
•Child-Pugh score B or C	6 (38)	4 (67)	1 (50)		0.5589
•MELD score, mean ± SD	9.7 ± 2.9	12.3 ± 3.9	12.5 ± 0.7		0.1793
Albumin (< 30 g/L) n (%)	12 (3)	5 (11)	1 (3)	1 (6)	**0.0415**
Prothrombin time (≤70%) n (%)	45 (10)	11 (22)	6 (14)	6 (33)	**0.0037**
AST (> 5 x ULN) n (%)	28 (6)	4 (8)	3 (7)	2 (12)	0.4951
ALT (> 5 x ULN) n (%)	31 (6)	2 (4)	1 (2)	1 (6)	0.7735
Haemoglobin (≤12 g/dL in women or ≤ 13 g/dL in men) n (%)	58 (12)	8 (16)	5 (11)	1 (6)	0.7365
Platelets < 100,000/mm^3^	87 (19)	15 (30)	11 (25)	8 (47)	**0.0109**
Bilirubin conj ≥5 μmol/L	133 (52)	20 (63)	16 (57)	13 (100)	**0.0021**
Treatment history n (%)					
•Naïve patients	141 (29)	13 (25)	14 (31)	5 (28)	0.0618
•Experienced patients, last treatment PEG/RBV	324 (67)	35 (69)	25 (56)	10 (56)	
•Experienced patients, last treatment 1rst generation PI/PEG/RBV	20 (4)	3 (6)	6 (13)	3 (17)	
Response profile in treatment experienced patients					
•Unknown	164 (48)	20 (53)	17 (55)	6 (46)	0.8370
•Responders	86 (25)	9 (24)	4 (13)	3 (23)	
•Not responders	94 (27)	9 (24)	10 (32)	4 (31)	

*SOF* Sofosbuvir, *SMV* Simeprevir, *RBV* Ribavirine, *PI* Protease inhibitor, *SD* Standard deviation, *BMI* Body mass index

*P* value inferior to 0.05 are in boldface

### Efficacy

Missing SVR12 measurements were determined using SVR24 measurements in 52 patients and SVR4 measurements in 6 patients. Four patients who died before reaching SVR12 and seven patients who were lost to follow-up with no virological response measurement at the end of therapy were considered virological failures.

**Fig. 2:**
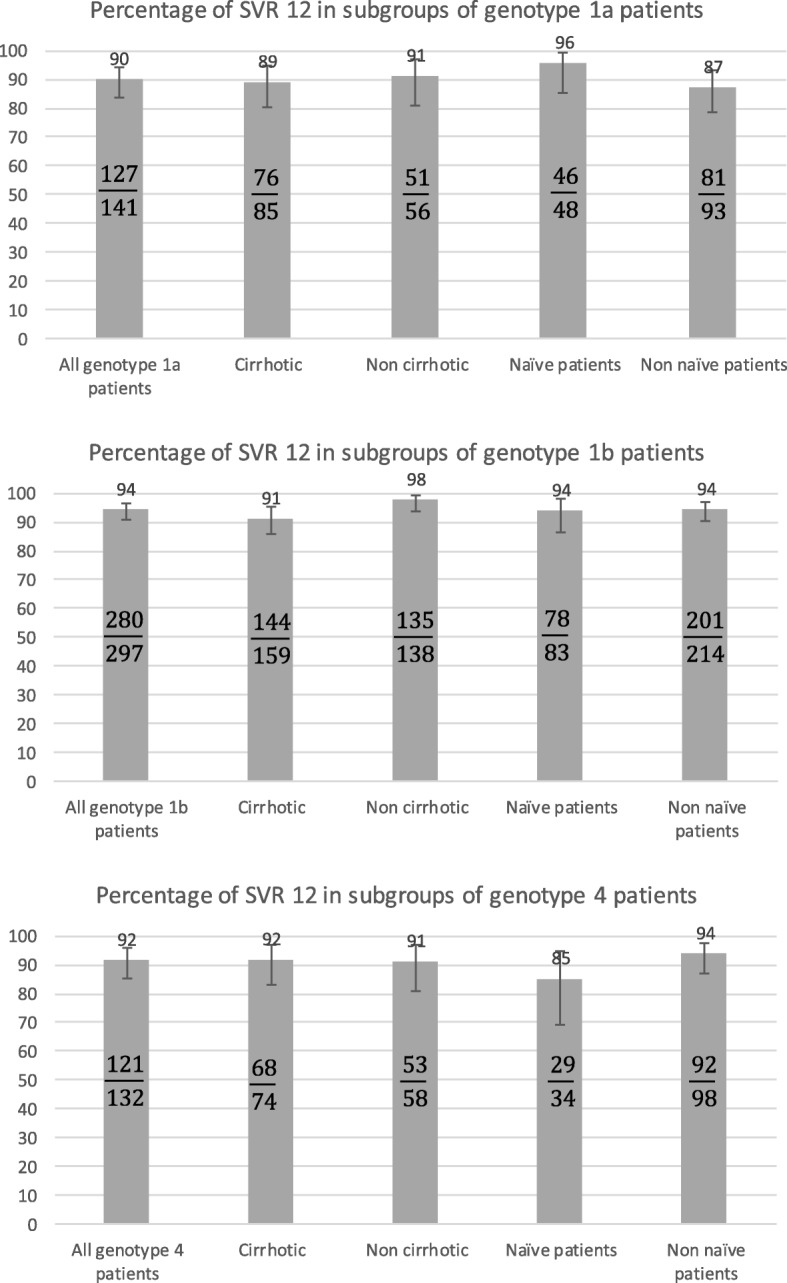
Percentages of SVR12 in subgroups of patients by genotype

A SVR12 was achieved in a total of 555 (92.6%) patients. SVR12 rates ranged from 89% in patients who received a 24-week sofosbuvir/simeprevir/ribavirin combination regimen to 98% in patients who received a 12-week sofosbuvir/simeprevir/ribavirin combination regimen (Table 2 and Fig. 2).

**Table 2:** Virologic responses according to therapeutic regimens

	TOTAL *N* = 599	Sofosbuvir + simeprevir		Sofosbuvir + simeprevir + ribavirin		Fisher *p* value	CMH *p* value (strati-fication: 12 weeks vs 24 weeks)	CMH *p* value (strati-fication: RBV vs no RBV)
		12 weeks n = 485	24 weeks n = 51	12 weeks n = 45	24 weeks n = 18			
Negative HCV RNA								
Week 12 n/N (%)	519/576 (90)	422/469 (90)	41/47 (87)	43/44 (98)	13/16 (81)	0.1312	0.2779	0.1514
Week 24n/N (%)	60/65 (92)		44/49 (90)		16/16 (100)	0.3219	0.1869	
Follow up week 4n/N (%)	277/570 (49)	318/341 (93)	30/31 (97)	39/40 (98)	13/13 (100)	0.7525	0.2341	0.3669
SVR 12 (imputed^a^) n/N (%)	555/599 (93)	449/485 (93)	46/51 (90)	44/45 (98)	16/18 (89)	0.3742	0.3197	0.2538
SVR 24 n/N (%)	459/473 (97)	374/385 (97)	38/40 (95)	37/38 (97)	10/10 (100)	0.7360	0.6713	0.6102
SVR 12 in non cirrhotic patients n/N (%)	244/257 (95)	206/219 (94)	18/18 (100)	18/18 (100)	2/2 (100)	0.5937	0.2887	0.2887
SVR 12 in cirrhotic patients n/N (%)	304/335 (91)	236/259 (91)	28/33 (85)	26/27 (96)	14/16 (88)	0.4043	0.3882	0.1293
SVR 12 in treatment naïve patientsn/N (%)	160/173 (92)	130/141 (92)	12/13 (92)	14/14 (100)	4/5 (80)	0.3637	0.6195	0.4809
SVR 12 in treatment experienced patients n/N (%)	395/426 (93)	319/344 (93)	34/38 (89)	30/31 (97)	12/13 (92)	0.6749	0.3874	0.3635
Last treatment								
PEG/RBV n/N (%)	365/394 (93)	300/324 (93)	32/35 (91)	24/25 (96)	9/10 (90)	0.8073	0.6462	0.6214
First generation PI/PEG/RBV n/N (%)	30/32 (94)	19/20 (95)	2/3 (67)	6/6 (100)	3/3 (100)	0.3750	0.2637	0.1122
Response profile								
Not respondersn/N (%)	108/117 (92)	87/94 (93)	8/9 (89)	9/10 (90)	4/4 (100)	0.6151	0.9339	0.9864
Responders^b^ n/N (%)	99/102 (97)	84/86 (98)	8/9 (89)	4/4 (100)	3/3 (100)	0.4040	0.5154	0.1537
Unknown n/N (%)	188/207 (91)	148/164 (90)	18/20 (90)	17/17 (100)	5/6 (83)	0.4343	0.3647	0.5569
SVR 12 in Cirrhotic Treatment experienced patients n/N(%)	230/258 (89)	178/200 (89)	23/27 (85)	18/19 (95)	11/12 (92)	0.8219	0.3407	0.5044
SVR 12 in patients with genotype 1a n/N (%)	127/141 (90)	99/110 (90)	10/11 (91)	13/14 (93)	5/6 (83)	0.8660	0.9608	0.7862
SVR 12 in patients with genotype 1b infection n/N (%)	280/297 (94)	237/251 (94)	20/22 (91)	18/18 (100)	5/6 (83)	0.2403	0.6111	0.2015
SVR 12 in patients with genotype 4 infection n/N (%)	121/132 (92)	89/99 (90)	16/17 (94)	10/10 (100)	6/6 (100)	0.9150	0.2283	0.5850
SVR 12 in patients with genotype 1 not subtyped n/N (%)	27/29 (93)	24/25 (96)	0/1 (0)	3/3 (100)		0.0764	0.7290	0.0005

*HCV* Hepatitis C Virus, *SVR* Sustain virological response, *PI* Protease inhibitor, *RBV* Ribavirin, *PEG* Pegylated interferon.

^a^imputed: missing SVR12 measurements were imputed using SVR24 measurement if available (n = 52), otherwise using SVR4 measurement (n = 6). We imputed a virological failure in patients who died before SVR12 and therefore could not be evaluated (n = 4) and in patients who were lost of follow up (n = 7) and had no measurement of the virological response after end of therapy. ^b^ responders = patients with negative HCV RNA on last treatment - includes one patient with sustained virological response who was re-infected

Although no significant difference was found between the 12- or 24-week regimen with or without ribavirin, the groups were not comparable because patients in the ribavirin and 24-week treatment groups had more severe disease.

The SVR12 rate in patients without cirrhosis was 94.9%, whatever the treatment.

The SVR12 rate in patients with cirrhosis was 90.7% in treatment-naïve and 89% in treatment-experienced patients. No significant difference was found between those who received a 24-week regimen and a 12-week regimen (42/49 (85.7% %) versus 262/286 (91.6%), respectively (CMH Chi Square stratified on ribavirin containing regimen: *p* = 0.12)). The SVR12 rate was not different between those who received a 12-week regimen with or without ribavirin (26/27 (96%) versus 236/259 (91%), respectively (CMH Chi Square stratified on ribavirin containing regimen: *p* = 0.39)).

The overall SVR12 was 90, 94.2 and 91.6% in patients with genotypes 1a, 1b and 4, respectively, and was not significant different among the groups (Table 3). The addition of ribavirin or not, and the duration of treatment (12 vs 24 weeks) did not influence the SVR12 rate.

**Table 3:** Variables associated with SVR12 in univariate and multivariate analysis

	n with SVR 12 / Total (%): Yes VS Reference	Univariate		Multivariate	
		OR (95% CI)	*p*-Value	OR (95% CI)	*p*-Value
Ribavirin containing regimen (reference: no RBV)	60/63 (95) VS 495/536 (92)	1.66 (0.5–8.61)	0.5936		
Treatment duration 24 weeks (reference: 12 weeks)	62/69 (90) VS 493/530 (93)	0.67 (0.28–1.85)	0.4654		
Cirrhosis (reference: no cirrhosis)	304/335 (91) VS 244/257 (95)	0.52 (0.25–1.06)	0.0734	1.07 (0.36–3.20)	1.0000
Conjugated bilirubin ≥5 μmol/L (reference: < 5 μmol/L)	163/182 (90) VS 141/148 (95)	0.43 (0.15–1.1)	0,0835	0.58 (0.19–1.67)	0.3701
TP ≤ 70% (reference: > 70%)	59/68 (87) VS 458/491 (93)	0.47 (0.21–1.18)	0.1103	0.41 (0.14–1.22)	0.1148
Hemoglobin ≤12 g/dL for women or ≤ 13 g/dL for men(reference: > 12 g/dL or > 13 g/dL)	63/72 (88) VS 480/514 (93)	0.5 (0.22–1.23)	0.1338	0.59 (0.22–1.71)	0.3620
Platelets < 100,000/mm^3^ (reference: ≥ 100,000/mm^3^)	108/121 (89) VS 429/458 (94)	0.56 (0.27–1.22)	0.1505	1.12 (0.39–3.38)	1.0000
Genotype 1b (reference: 1 not subtyped or 1a or 4)	280/297 (94) VS 275/302 (91)	1.62 (0.83–3.24)	0.1757	0.94 (0.39–2.26)	1.0000
Male (reference: Female)	303/322 (94) VS 252/277 (91)	1.18 (0.65–2.13)	0.6504	1.62 (0.68–3.94)	0.3192
Genotype 1a (reference: 1 not subtyped or 1b or 4)	127/141 (90) VS 428/458 (93)	0.64 (0.32–1.34)	0.2489		
Neutrophil < 1500/mm^3^ (reference: ≥ 1500/mm^3^)	33/38 (87) VS 503/540 (93)	0.49 (0.17–1.69)	0.2641		
Chronic hepatitis duration ≥15 years (reference: < 15 years)	308/337 (91) VS 236/251 (94)	0.68 (0.33–1.34)	0.2980		
Albumin < 30 g/L (reference: ≥ 30 g/L)	16/19 (84) VS 479/519 (92)	0.45 (0.12–2.49)	0.3756		
Age > 65 years (reference: ≤ 65 years)	209/223 (94) VS 346/376 (92)	1.29 (0.65–2.7)	0.5478		
MELD ≥15 (reference: < 15)	31/35 (89) VS 454/491 (92)	0.63 (0.21–2.6)	0.5732		
Genotype 4 (reference: 1a or 1b or 1 not subtyped)	121/132 (92) VS 434/467 (93)	0.84 (0.4–1.89)	0.7409		
Viral load < 6 M at beginning of treatment (reference: ≥ 6 M)	502/542 (93) VS 42/44 (95)	0.6 (0.07–2.45)	0.7425		
ALAT > 5 ULN (reference: ≤ 5ULN)	33/35 (94) VS 511/551 (93)	1.29 (0.31–11.5)	0.9999		
ASAT > 5 ULN (reference: ≤ 5ULN)	34/37 (92) VS 508/547 (93)	0.87 (0.26–4.63)	0.9999		
Decompensated cirrhosis (reference: no decompensated cirrhosis)	22/24 (92) VS 533/575 (93)	0.87 (0.2–7.86)	0.9999		
Treatment experienced (reference: treatment naïve)	160/173 (92) VS 395/426 (93)	0.97 (0.48–2.07)	0.9999		
Genotype 1 not subtyped (reference: 1a or 1b or 4)	27/29 (93) VS 528/570 (93)	1.07 (0.25–9.63)	0.9999		
Previous treatment: PEG/RBV (reference: 1st generation PI/PEG/RBV or none)	365/394 (93) VS 190/205 (93)	0.99 (0.48–1.97)	0.9999		

*SVR* Sustain virological response, *RBV* Ribavirin, *PEG* Pegylated interferon, *PI* Protease inhibitor

There were no variables associated with SVR12 at the 0.05 level on univariate analysis (Table 3).

Age, sex, gender, BMI, genotype, prior treatment history, cirrhosis or not and treatment duration did not influence SVR12. No factor was associated with the absence of SVR12 on multivariate analysis.

### Safety and tolerability

Early treatment discontinuation only occurred in 18 patients (3%). The rate of discontinuation for adverse events was 1.5%. The rate of discontinuation was higher in patients treated for 24 weeks or with ribavirin (Table 4). Nine of the patients who discontinued treatment (50%) achieved a SVR12. Eight (44%) had been treated for 8 weeks or more.

**Table 4:** Adverse events

	Sofosbuvir + simeprevir		Sofosbuvir + simeprevir+ ribavirin		Fisher *P*-value	CMH *P* value (stratification: 12 weeks vs 24 weeks)	CMH *P* value (stratification: RBV vs no RBV)
	12 weeks	24 weeks	12 weeks	24 weeks			
Number of patients	485	51	45	18			
Treatment interruptions n (%)	6 (1)	4 (8)	5 (11)	3 (17)	0.0000	0.0003	0.0130
‐ Adverse event	3 (1)	2 (4)	3 (7)	1 (6)	0.0030	0.0074	0.2081
‐ Other reasons	3 (1)	2 (4)	2 (4)	2 (11)	0.0014	0.0169	0.0300
All adverse events - any n (%)	312 (64)	35 (69)	32 (71)	15 (83)	0.3185	0.1720	0.3247
(Maximum grade)							
‐ Grade 1	156 (32)	15 (29)	9 (20)	3 (17)	< 0.0001	0.0043	< 0.0001
‐ Grade 2	116 (24)	7 (14)	19 (42)	10 (56)			
‐ Grade 3	28 (6)	3 (6)	2 (4)	0 (0)			
‐ Grade 4	11 (2)	8 (16)	2 (4)	1 (6)			
‐ Grade 5	1 (0)	2 (4)	0 (0)	1 (6)			
Deaths	1 (0)	2 (4)	0 (0)	1 (6)	0.0096	0.8684	0.0003
Other serious adverse events	26 (5)	7 (14)	3 (7)	1 (6)	0.1241	0.7697	0.0453
Adverse Events (≥10% in any subgroup)							
‐ Asthenia	80 (16)	11 (22)	17 (38)	4 (22)	0.0074	0.0023	0.9114
‐ Headache	69 (14)	2 (4)	3 (7)	4 (22)	0.0457	0.7803	0.2836
‐ Pruritus	42 (9)	1 (2)	3 (7)	3 (17)	0.1653	0.5673	0.3966
‐ Hyperbilirubinaemia	28 (6)	5 (10)	7 (16)	5 (28)	0.0015	0.0018	0.1129
‐ Fatigue	33 (7)	2 (4)	0 (0)	2 (11)	0.1531	0.2982	0.9563
‐ Thrombocytopenia	20 (4)	7 (14)	2 (4)	3 (17)	0.0059	0.7737	0.0007
‐ Insomnia	24 (5)	2 (4)	3 (7)	2 (11)	0.4667	0.3140	0.9557
‐ Sleep disorder	21 (4)	2 (4)	1 (2)	3 (17)	0.1160	0.6041	0.3077
‐ Dry skin	7 (1)	0 (0)	1 (2)	2 (11)	0.0526	0.0711	0.6618
‐ Oedema peripheral	4 (1)	2 (4)	0 (0)	2 (11)	0.0117	0.5464	0.0028
‐ Eczema	3 (1)	2 (4)	0 (0)	2 (11)	0.0059	0.4760	0.0012
‐ Dyspnoea	1 (0)	2 (4)	1 (2)	2 (11)	0.0004	0.0586	0.0019
‐ Gastrointestinal disorder	4 (1)	0 (0)	0 (0)	2 (11)	0.0336	0.1701	0.2244
‐ Jaundice	0 (0)	1 (2)	2 (4)	2 (11)	0.0001	0.0003	0.0579

*RBV* Ribavirin

Four patients died during follow-up. One patient died from a subdural hematoma in the first week after initiating treatment, and one patient at week 8 from undetermined causes. Two patients died suddenly at week 12 from cardiac arrest, which was considered to be possibly treatment-related (sofosbuvir/simeprevir). Cardiovascular side effects (mainly bradyarrhytmias) have been associated with sofosbuvir treatment and may result in the implementation of a pace-maker [35] and associated with a risk of sudden, unexplained death [36].

Forty-three other serious adverse events occurred in 37 (9%) patients with no difference between treatment with or without ribavirin, but with a higher rate in the 24- week regimen (*p* = 0.0453). Two of these serious adverse events were considered to be possibly treatment-related (simeprevir): one malaise at week 12 and one drug-induced acute hepatitis at week 5.

The most common adverse events (≥10% in any subgroup) were asthenia, headache and pruritus.

Univariate analysis identified treatment duration, prothrombin time ≤ 70%, decompensated cirrhosis, time since diagnosis ≥15 years, MELD ≥15 or cirrhosis at inclusion, cirrhosis, platelet count < 100,000/mm^3^, conjugated bilirubin ≥5 μmol/L and albumin < 30 g/L as potential predictors of serious adverse events. A prothrombin time ≤ 70% (OR versus prothrombin time ≥ 70%, 2.88 95%CI 1.24–6.48; *P* = 0.0127), MELD ≥15 or cirrhosis (OR versus MELD < 15 and no cirrhosis, 3.13 95%CI 1.2–9.62; *P* = 0.0154) and a time since diagnosis ≥15 years (OR versus time since diagnosis < 15 years, 2.19 95%CI 1.01–5.1; *P* = 0.0465) remained the only 3 factors independently associated with serious adverse events. It should be noted that age and gender were not associated with serious adverse events.

## Discussion

Although the real-life results of the sofosbuvir/simeprevir combination have been extensively reported in US genotype 1-infected patients, data from other geographical areas or other genotypes are limited. In this real-life study, we analyzed the efficacy and safety of the sofosbuvir+simeprevir +/− ribavirin combination in patients with genotypes 1 or 4 infection from the French ANRS CO22 HEPATHER cohort, a real-life study. Most of these patients were “difficult-to-treat” since 56% had cirrhosis, 4% had decompensated cirrhosis, 71% had failed prior treatment with pegylated interferon and ribavirin and 5% associated with telaprevir or boceprevir. Only 7 patients (1.16%) were lost to follow-up with no available PCR after the end of treatment. The sofosbuvir/simeprevir combination resulted in a global SVR12 rate of 92.6%. Most patients (81%) received the sofosbuvir/simeprevir combination without ribavirin for 12 weeks with a SVR of 93%. No significant difference in SVR12 rate was found between 12 or 24 weeks of treatment, with or without ribavirin. However, it is not possible to conclude whether extending the duration of treatment or the addition of ribavirin is needed or not, especially in patients with cirrhosis or decompensated cirrhosis because of the small sample size and because patients had more severe liver disease in the ribavirin and 24- week regimen groups. The overall SVR12 was 94.2% in patients with genotype 1b infection (vs 90 and 91.6% for genotypes 1a and 4, respectively) but the difference was not statistically significant. There was no factor associated with treatment failure on univariate analysis.

Early treatment discontinuation was rare and no new safety signals were reported compared to previous studies. The severity of liver disease (MELD ≥15 or cirrhosis at inclusion) was a risk factor for serious adverse events which support a causal relationship between adverse events and protease inhibitor exposure, as previously reported in the CUPIC study [37].

It is not possible to compare our results with those of other studies, clinical trials or real-world studies, because the rate of “difficult to treat” patients differed. The SVR12 rate in the real-world US TARGET cohort [25] including 59% of patients with cirrhosis (56% in our study) and around 50% of treatment-experienced patients (71% in our study) was 84%. In that study, in contrast to our results, the severity of liver disease and previous protease inhibitor treatments were associated with treatment failure in the model-adjusted estimates. In an Egyptian [32] and a US real-life study [38] the SVR rate was > 92%. In summary, our study shows a SVR-rate of nearly 95% in patients without cirrhosis, and 91% in those with cirrhosis, which is comparable to other real-life studies [31–33].

Even with a SVR rate of 91–95%, the role of simeprevir/sofosbuvir, is debatable with the current high turnover of DAAs. In the era of “second” wave DAAs (sofosbuvir with ledipasvir, daclatasvir or velpatasvir and voxilaprevir, ombitasvir/paritaprevir/dasabuvir, grazoprevir/elbasvir, glecaprevir/pibrentasvir) with high SVR rates in both clinical trials and real-life studies [34] and access to shorter-duration pangenotypic regimens even in patients with cirrhosis, our real-world study suggests that the efficacy of the 12-week sofosbuvir/simeprevir combination is probably suboptimal despite an acceptable safety profile.

## Conclusion

This study reports the real-life results of the French ANRS CO22 Hepather cohort for the sofosbuvir+simeprevir +/− ribavirin combination in patients with HCV genotypes 1 or 4 mono-infection. The overall SVR12 was 92.6, 90% in patients with genotype 1a infection, 94.2% with genotype 1b and 91.6% with genotype 4 with an acceptable safety profile. In the era of “second” wave DAAs this combination is no longer recommended in the most recent (2018) EASL guidelines but could remain a therapeutic option in low-income countries without access to pangenotypic drugs.

## Abbreviations

BMI: Body mass index
DAA: Direct-acting antiviral
EASL: European Association for the Study of the Liver
HCV: Hepatitis C virus
PCR: Polymerase chain reaction
PEG: Pegylated interferon
PI: Protease inhibitor
RBV: Ribavirin
RNA: Ribonucleic acid
SD: Standard deviation
SMV: Simeprevir
SOF: Sofosbuvir
SVR: Sustained virological response

## References

[1] Younossi ZM, Kanwal F, Saab S, Brown KA, El-Serag HB, Kim WR (2014). The impact of hepatitis C burden: an evidence-based approach. Aliment Pharmacol Ther.

[2] Marcellin P, Pequignot F, Delarocque-Astagneau E, Zarski JP, Ganne N, Hillon P (2008). Mortality related to chronic hepatitis B and chronic hepatitis C in France: evidence for the role of HIV coinfection and alcohol consumption. J Hepatol.

[3] Deuffic-Burban S, Deltenre P, Louvet A, Canva V, Dharancy S, Hollebecque A (2008). Impact of viral eradication on mortality related to hepatitis C: a modeling approach in France. J Hepatol.

[4] Backus LI, Boothroyd DB, Phillips BR, Belperio P, Halloran J, Mole LA (2011). A sustained virologic response reduces risk of all-cause mortality in patients with hepatitis C. Clin Gastroenterol Hepatol.

[5] van der Meer AJ, Veldt BJ, Feld JJ, Wedemeyer H, Dufour JF, Lammert F (2012). Association between sustained virological response and all-cause mortality among patients with chronic hepatitis C and advanced hepatic fibrosis. Jama..

[6] Nahon P, Bourcier V, Layese R, Audureau E, Cagnot C, Marcellin P (2017). Eradication of hepatitis C virus infection in patients with cirrhosis reduces risk of liver and non-liver complications. Gastroenterology.

[7] Sofia MJ, Bao D, Chang W, Du J, Nagarathnam D, Rachakonda S (2010). Discovery of a beta-d-2′-deoxy-2′-alpha-fluoro-2′-beta-C-methyluridine nucleotide prodrug (PSI-7977) for the treatment of hepatitis C virus. J Med Chem.

[8] Raboisson P, de Kock H, Rosenquist A, Nilsson M, Salvador-Oden L, Lin TI (2008). Structure-activity relationship study on a novel series of cyclopentane-containing macrocyclic inhibitors of the hepatitis C virus NS3/4A protease leading to the discovery of TMC435350. Bioorg Med Chem Lett.

[9] Penin F, Dubuisson J, Rey FA, Moradpour D, Pawlotsky JM (2004). Structural biology of hepatitis C virus. Hepatology (Baltimore, Md).

[10] Lawitz E, Mangia A, Wyles D, Rodriguez-Torres M, Hassanein T, Gordon SC (2013). Sofosbuvir for previously untreated chronic hepatitis C infection. N Engl J Med.

[11] Lawitz E, Sulkowski MS, Ghalib R, Rodriguez-Torres M, Younossi ZM, Corregidor A (2014). Simeprevir plus sofosbuvir, with or without ribavirin, to treat chronic infection with hepatitis C virus genotype 1 in non-responders to pegylated interferon and ribavirin and treatment-naive patients: the COSMOS randomised study. Lancet (London, England).

[12] Gane EJ, Stedman CA, Hyland RH, Ding X, Svarovskaia E, Symonds WT (2013). Nucleotide polymerase inhibitor sofosbuvir plus ribavirin for hepatitis C. N Engl J Med.

[13] Sulkowski MS, Gardiner DF, Rodriguez-Torres M, Reddy KR, Hassanein T, Jacobson I (2014). Daclatasvir plus sofosbuvir for previously treated or untreated chronic HCV infection. N Engl J Med.

[14] Kowdley KV, Gordon SC, Reddy KR, Rossaro L, Bernstein DE, Lawitz E (2014). Ledipasvir and sofosbuvir for 8 or 12 weeks for chronic HCV without cirrhosis. N Engl J Med.

[15] Kowdley KV, Lawitz E, Poordad F, Cohen DE, Nelson DR, Zeuzem S (2014). Phase 2b trial of interferon-free therapy for hepatitis C virus genotype 1. N Engl J Med.

[16] Zeuzem S, Jacobson IM, Baykal T, Marinho RT, Poordad F, Bourliere M (2014). Retreatment of HCV with ABT-450/r-ombitasvir and dasabuvir with ribavirin. N Engl J Med.

[17] Kwo P, Gitlin N, Nahass R, Bernstein D, Etzkorn K, Rojter S, et al. Simeprevir plus Sofosbuvir (12 and 8 weeks) in HCV genotype 1-infected patients without cirrhosis: OPTIMIST-1, a phase 3, randomized study. Hepatology (Baltimore, Md). 2016;64(2):370–80. 10.1002/hep.28467 PMC5412860 26799692

[18] Lawitz E, Matusow G, DeJesus E, Yoshida EM, Felizarta F, Ghalib R, et al. Simeprevir plus sofosbuvir in patients with chronic hepatitis C virus genotype 1 infection and cirrhosis: a phase 3 study (OPTIMIST-2). Hepatology (Baltimore, Md). 2015;64(2):360–69. 10.1002/hep.28422 PMC5297873 26704148

[19] Kwo PY, Poordad F, Asatryan A, Wang S, Wyles DL, Hassanein T (2017). Glecaprevir and pibrentasvir yield high response rates in patients with HCV genotype 1-6 without cirrhosis. J Hepatol.

[20] Feld JJ, Jacobson IM, Hezode C, Asselah T, Ruane PJ, Gruener N (2015). Sofosbuvir and Velpatasvir for HCV genotype 1, 2, 4, 5, and 6 infection. N Engl J Med.

[21] Bourliere M, Gordon SC, Flamm SL, Cooper CL, Ramji A, Tong M (2017). Sofosbuvir, Velpatasvir, and Voxilaprevir for previously treated HCV infection. N Engl J Med.

[22] EASL (2015). Recommendations on treatment of hepatitis C 2015. J Hepatol.

[23] AASLD. HCV guidance : Recommendations for testing, managing, and treating hepatitis C. 2015.

[24] EASL (2018). Recommendations on treatment of hepatitis C 2018. J Hepatol.

[25] Sulkowski MS, Vargas HE, Di Bisceglie AM, Kuo A, Reddy KR, Lim JK (2016). Effectiveness of Simeprevir plus Sofosbuvir, with or without ribavirin, in real-world patients with HCV genotype 1 infection. Gastroenterology..

[26] Shiffman ML, James AM, Long AG, Alexander PC (2015). Treatment of chronic HCV with sofosbuvir and simeprevir in patients with cirrhosis and contraindications to interferon and/or ribavirin. Am J Gastroenterol.

[27] Saxena V, Nyberg L, Pauly M, Dasgupta A, Nyberg A, Piasecki B (2015). Safety and efficacy of Simeprevir/Sofosbuvir in hepatitis C-infected patients with compensated and decompensated cirrhosis. Hepatology (Baltimore, Md).

[28] Backus LI, Belperio PS, Shahoumian TA, Loomis TP, Mole LA (2015). Effectiveness of sofosbuvir-based regimens in genotype 1 and 2 hepatitis C virus infection in 4026 U.S. veterans. Aliment Pharmacol Ther.

[29] Aqel BA, Pungpapong S, Leise M, Werner KT, Chervenak AE, Watt KD (2015). Multicenter experience using simeprevir and sofosbuvir with or without ribavirin to treat hepatitis C genotype 1 in patients with cirrhosis. Hepatology (Baltimore, Md).

[30] Dieterich D, Bacon BR, Flamm SL. Evaluation of sofosbuvir and simeprevir-based regimens in the trio network: academic and community of a real-world, heterogenous population. Hepatology (Baltimore, Md). 2014;60(Suppl 1):220A–220A.

[31] Willemse SB, Baak LC, Kuiken SD, van der Sluys VA, Lettinga KD, van der Meer JT, et al. Sofosbuvir plus simeprevir for the treatment of HCV genotype 4 patients with advanced fibrosis or compensated cirrhosis is highly efficacious in real life. J Viral Hepat. 2016;1–5. 10.1111/jvh.12567 27405785

[32] El-Khayat HR, Fouad YM, Maher M, El-Amin H, Muhammed H (2017). Efficacy and safety of sofosbuvir plus simeprevir therapy in Egyptian patients with chronic hepatitis C: a real-world experience. Gut..

[33] Marino Z, Pascasio-Acevedo JM, Gallego A, Diago M, Baliellas C, Morillas R (2017). High efficacy of Sofosbuvir plus Simeprevir in a large cohort of Spanish cirrhotic patients infected with genotypes 1 and 4. Liver Int.

[34] Pol S, Bourliere M, Lucier S, Hezode C, Dorival C, Larrey D (2017). Safety and efficacy of daclatasvir-sofosbuvir in HCV genotype 1-mono-infected patients. J Hepatol.

[35] Fontaine H, Lazarus A, Pol S, Pecriaux C, Bagate F, Sultanik P (2015). Bradyarrhythmias associated with Sofosbuvir treatment. N Engl J Med.

[36] Laurain A, Kramer L, Sultanik P, Vallet-Pichard A, Sogni P, Pol S (2018). Mortality associated with the treatment of HCV with direct-acting antivirals. Gut.

[37] Hezode C, Fontaine H, Dorival C, Larrey D, Zoulim F, Canva V (2013). Triple therapy in treatment-experienced patients with HCV-cirrhosis in a multicentre cohort of the French early access Programme (ANRS CO20-CUPIC) - NCT01514890. J Hepatol.

[38] Alam I, Brown K, Donovan C, Forlenza J, Lauwers K, Mah'moud MA (2017). Real-world effectiveness of Simeprevir-containing regimens among patients with chronic hepatitis C virus: the SONET study. Open Forum Infect Dis.

